# Binational Arsenic Exposure Survey: Methodology and Estimated Arsenic Intake from Drinking Water and Urinary Arsenic Concentrations

**DOI:** 10.3390/ijerph9041051

**Published:** 2012-03-26

**Authors:** Jason Roberge, Mary Kay O’Rourke, Maria Mercedes Meza-Montenegro, Luis Enrique Gutiérrez-Millán, Jefferey L. Burgess, Robin B. Harris

**Affiliations:** 1 Department of Epidemiology and Biostatistics, Mel and Enid Zuckerman College of Public Health, The University of Arizona, 1515 N. Campbell Ave., Tucson, AZ 85724, USA; Email: rharris@azcc.arizona.edu; 2 Division of Community, Environment and Policy, Mel and Enid Zuckerman College of Public Health, The University of Arizona, Tucson, AZ 85724, USA; Email: mkor@email.arizona.edu (M.K.O’R.); jburgess@email.arizona.edu (J.L.B.); 3 Department of Biotechnology and Food Sciences, Instituto Tecnológico de Sonora, 5 de Febrero 818 Sur, Zona Centro, Cd. Obregon, Sonora 85000, Mexico; Email: mmeza@itson.mx; 4 Department of Scientific and Technological Research, University of Sonora, Hermosillo, Sonora 83000, Mexico; Email: legtz@guaymas.uson.mx

**Keywords:** arsenic, urine, water, beverages, metabolite, intake, BAsES

## Abstract

The Binational Arsenic Exposure Survey (BAsES) was designed to evaluate probable arsenic exposures in selected areas of southern Arizona and northern Mexico, two regions with known elevated levels of arsenic in groundwater reserves. This paper describes the methodology of BAsES and the relationship between estimated arsenic intake from beverages and arsenic output in urine. Households from eight communities were selected for their varying groundwater arsenic concentrations in Arizona, USA and Sonora, Mexico. Adults responded to questionnaires and provided dietary information. A first morning urine void and water from all household drinking sources were collected. Associations between urinary arsenic concentration (total, organic, inorganic) and estimated level of arsenic consumed from water and other beverages were evaluated through crude associations and by random effects models. Median estimated total arsenic intake from beverages among participants from Arizona communities ranged from 1.7 to 14.1 µg/day compared to 0.6 to 3.4 µg/day among those from Mexico communities. In contrast, median urinary inorganic arsenic concentrations were greatest among participants from Hermosillo, Mexico (6.2 µg/L) whereas a high of 2.0 µg/L was found among participants from Ajo, Arizona. Estimated arsenic intake from drinking water was associated with urinary total arsenic concentration (*p* < 0.001), urinary inorganic arsenic concentration (*p* < 0.001), and urinary sum of species (*p* < 0.001). Urinary arsenic concentrations increased between 7% and 12% for each one percent increase in arsenic consumed from drinking water. Variability in arsenic intake from beverages and urinary arsenic output yielded counter intuitive results. Estimated intake of arsenic from all beverages was greatest among Arizonans yet participants in Mexico had higher urinary total and inorganic arsenic concentrations. Other contributors to urinary arsenic concentrations should be evaluated.

## 1. Introduction

Arsenic (As) is a widely distributed element found naturally in the Earth’s crust [1]. Arsenic is classified as a known human carcinogen and is among the top 20 hazardous materials compiled by the Environmental Protection Agency (EPA) [2,3]. Consumption of As^3^ and As^5^ at levels above 100 µg/L over long periods of time has been associated with lung, bladder, and skin cancer [4,5,6]. 

The health effects of arsenic consumption are attributed primarily to exposures from higher arsenic concentrations (>100 µg/L) found in local water supplies [4,6,7,8,9,10]. Yet, the contribution of arsenic from foods and beverages may play a larger role than arsenic from regulated drinking water in defining the total arsenic concentration in urine [11,12]. This may be the case particularly where populations are consuming relatively lower concentrations of arsenic from drinking water. Few studies have examined the effects of the consumption of arsenic from drinking water at the maximum contaminant level, 10 µg/L, established by the United States EPA at in 2006 [13].

In the United States (U.S.), arsenic concentration in groundwater is greatest in the West, although isolated drainage basins in the Midwest and Northeast exceed 10 µg/L is some areas. Public water utilities are mandated to have no more than 10 µg/L of arsenic in water supplied for domestic use. It is a public health concern that owners of private wells are not required to assess and control arsenic or other contaminants from their drinking and cooking water. Limited information is available about water consumption patterns among people in the U.S. and less is known for those in Mexico. National surveys inquire about water consumption, but few distinguish the various sources of water and other beverages consumed in the home.

The Binational Arsenic Exposure Survey (BAsES) was designed to assess arsenic concentrations in drinking water among selected communities in Arizona and Mexico and describe the relationships between estimated As concentrations in water and other beverages and As exposure reflected by urinary biomarkers. This pilot study also sought to build a binational research collaboration across the U.S.–Mexico border. Utilizing terminology as found in the International Society for Exposure Analysis glossary [14], the current study describes BAsES and examines the relationship between arsenic intake from water, other beverages, and urinary arsenic output.

## 2. Methods

### 2.1. Study Design

The Binational Arsenic Exposure Survey was cross-sectional in design and conducted in Arizona and northern Mexico. Communities in these regions were selected based on their arsenic concentrations contained in groundwater and likely represented a high versus low arsenic exposure among Mexicans, and Hispanic and Non-Hispanic participants within the U.S.

Randomly selected households were identified through phone interviews (e.g., random digit dialing) or door-to-door contact. All individuals who had lived in the home for at least one continuous year and were at least 18 years of age were eligible to participate. One person was selected randomly among household participants and identified as the primary respondent.

Interviewers from all sites received the same training regarding questionnaire administration. Interviews were administered in either English or Spanish as determined by the participant. The primary respondent completed a household questionnaire reporting characteristics of the home and individual respondents completed a personal questionnaire. In addition, biological samples, and environmental samples were collected during household visits. A single data entry system was created in Microsoft Access. De-identified data were electronically shared among the sites. The Arizona team served as the data coordinating center. Data were visually verified and outliers were evaluated.

Interviewing and sample collection protocols were reviewed by the Department of Health of Sonora in Mexico, respective university committees, and the Institutional Review Board (IRB) at the University of Arizona. All participants signed consent forms approved by the IRB in either Spanish or English. 

### 2.2. Recruitment—Arizona

In Arizona, 225 people from 152 households were recruited from the communities of Ajo, New River, San Manuel, and Tucson from January through October 2006. Data from Arizona were collected by personnel at the Mel and Enid Zuckerman College of Public Health and the Arizona Cancer Center at The University of Arizona. 

The goal was to recruit at least 25 households from each of the four communities. Recruitment primarily occurred through list-assisted random digit dialing. Up to 10 attempts were made to reach an individual at the designated phone number. The 2005–2006 QWEST phonebook was used for each Arizona community to gather phone pre-fixes and random phone numbers were generated.

An additional strategy was used in the rural community of New River. All residential addresses identified on rural delivery mailboxes were recorded and a recruitment letter was mailed to randomly selected addresses from the list. The letter contained a description of the study and a phone number to contact the study coordinator to schedule a date and time for consent and initial interview.

### 2.3. Recruitment—Mexico

In Mexico, 262 people were recruited from 202 households within two neighborhoods in Hermosillo and from the communities of Tobarito and Guadalupe Victoria in the Yaqui Valley near Ciudad Obregon. Recruitment occurred from January 2006 through February 2007 in Mexico. Data from Hermosillo were collected by personnel at the Universidad de Sonora (UNISON) and data from the Yaqui Valley were collected by personnel at the Instituto Tecnológico de Sonora (ITSON) in Ciudad Obregon.

**Figure 1:**
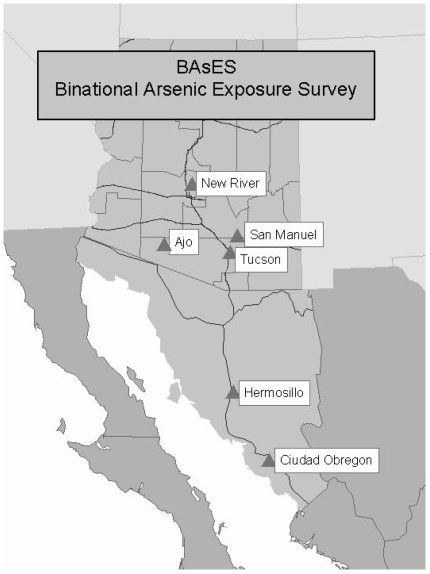
BAsES Recruitment Sites.

The aim was to recruit at least 50 households from each community or neighborhood. All wells supplying these neighborhoods/communities were tested for total arsenic to construct arsenic concentration maps. Each well supplied water to a defined neighborhood similar to a U.S. census tract. Seventy-three wells from the Yaqui Valley and 41 wells from Hermosillo were tested. In each region (Hermosillo and Yaqui Valley), one neighborhood with an elevated As concentration in the water supplied by the neighborhood well was selected and matched to another neighborhood with a lower As concentration in the water supply. Neighborhoods were matched on demographic characteristics (e.g., similar incomes, education level, *etc.*). The selected neighborhoods were subdivided into zones based on street boundaries or other map features and recruitment was conducted within a randomly selected zone. Every address within the boundary of the selected zone was recorded. Addresses excluded were clearly and exclusively non-residential. Addresses that were both a business and a residence were also included. Teams of interviewers went to randomly selected households within the recruitment zones to ask for participation. The recruitment sites are illustrated in Figure 1.

### 2.4. Household Questionnaire

The primary participant was asked to complete a household questionnaire containing questions about characteristics of the dwelling, availability of water, and access to a refrigerator, phone, computer and television.

### 2.5. Personal Questionnaires and Dietary History

All participants in the household were asked about demographics, smoking history, alcohol consumption, current occupation, job and hobby history, health history (e.g., history of diabetes, respiratory, cancers, *etc.*), medication and supplement use, reproductive history, physical activity, and residential history. 

Participants reported all water sources used for cooking and drinking in the home (kitchen tap water, bottle water, refrigerator spigot water, *etc.*) and frequency of use of each water source (frequently, moderately, rarely, never). The primary source of running water (well water, municipal water, *etc.*) and the presence of a water filtration device (reverse osmosis, charcoal filter, *etc.*) were recorded. 

Each participant completed a single 24 h administered dietary recall with portion size prompt using food models. Participants were asked to describe everything they ate or drank the previous day (including water) from the time they woke up in the morning until the time they went to bed (including anything eaten in the middle of the night). Interviewers recorded responses including amount consumed, specifics of preparation, and time of consumption.

The 24 h dietary recalls were sent to the Arizona Diet, Behavior and Quality of Life Assessment Lab (ADBA) at the Arizona Cancer Center for data entry and nutrient analysis using the Nutrition Data System for Research software version 2005 (NDSR), developed by the Nutrition Coordinating Center (NCC), University of Minnesota, Minneapolis, MN. Beverages (milk, apple juice, grape juice, *etc.*) were assigned total arsenic values as reported by Schoof *et al.* [15] USDA recipes were primarily used to estimate components of beverages that were not available in NDSR. Major components were then assigned arsenic values based on items reported by Schoof *et al.* [15].

The 24 h dietary recall quality control/quality assurance (QC/QA) procedures included a 15% duplicate entry of recalls by a different coder, followed by a comparison of entered recalls and reconciliation. Discrepancies attributable to coder error were addressed by closer surveillance when necessary. All data entry coders went through a formal training and probation period until their work met the ADBA standards. Whenever differing interpretations of a subject’s data set were identified, the recall was reviewed to provide consistency of coding.

### 2.6. Environmental and Biological Samples

Water, urine, and anthropometric measurements were collected from all participants. Water samples were collected from all sources of water reported as consumed in the home. An additional water sample was collected from outside the home (well, spigot, *etc.*) if a filtration system was present filtering the water coming into the home. 

Participants were supplied a urine cup and instructions for urine collection prior to the day of the interview. Participants were asked to collect a first morning urine sample. In the U.S., the urine cup was mailed to the participants. In Mexico, the urine cup was delivered by an interviewer the day before the interview. Specific gravity from the urine samples was determined using a refractometer.

Height, weight, hip and waist circumference were collected during the household interview. A calibrated digital scale was used to assess the weight of each participant in pounds. A stadiometer was used to assess the height of the participant. A standard measuring tape was used to assess hip and waist circumference. 

All samples were stored in a cooler on icepacks during transport from the interview site to the laboratory. Sample aliquots were stored at −80 °C. Samples of urine and water from Mexico were transported to The University of Arizona for the analysis of arsenic. Water samples were analyzed for total arsenic. Urine samples were analyzed for total arsenic, As^3^, As^5^, methylarsonic acid (MMA^5^), and dimethylarsinic acid (DMA^5^). All samples were analyzed for arsenic by the Southwest Hazardous Waste Program, Hazard Identification Core at The University of Arizona. Water and Urine samples were also analyzed for antimony, barium, beryllium, cadmium, cesium, cobalt, lead, molybdenum, platinum, selenium, thallium, tungsten, and uranium by the Bureau of State Laboratory Services at the Arizona Department of Health Services.

### 2.7. Laboratory Preparation and Analysis of Samples for Arsenic

Arsenic analyses were performed by the Analytical Section of the Hazard Identification Core from the University of Arizona. 

Preparation of urine samples for total arsenic analyses was accomplished by microwave digestion. A 1 mL aliquot of each beverage sample was placed in an acid-washed 7 mL teflon bomb, and 0.5 mL of concentrated nitric acid was added. Samples were sealed and heated in a microwave for 5 min on the medium-high setting. Samples were cooled, vented, and heated for an additional 5 min on the high setting. Additional steps of heating on the high setting were performed as necessary to complete sample digestion. After digestion, samples were brought up to volume with Milli-Q water for inductively coupled plasma mass spectroscopy (ICP-MS) analysis. Preparation of urine samples for arsenic speciation was accomplished by filtering the urine through 0.45 µm nylon centrifuge filters. 

Total arsenic concentrations in water and urine were determined using an Agilent 7500ce ICP-MS (Agilent Technologies, Inc., Santa Clara, CA) with a MicroMist nebulizer (Glass Expansion). An ASX500 auto sampler (CETAC Technologies) was used to introduce the samples into the Agilent 7500ce. The operating parameters were as follows: Rf power, 1500 watts; plasma gas flow, 15 L/min; carrier flow, 0.85 L/min; makeup gas, 0.15 L/min. Acquisition parameters were as follows: arsenic measured at *m/z* 75, terbium (internal standard) measured at *m/z* 159, points per peak 3, dwell time for arsenic was 1.5 s, and dwell time for terbium was 1.5 s. Each sample was evaluated seven times and the reported concentration represents the mean of these seven measures.

Concentrations of arsenic species in urine were analyzed using an Agilent 1100 HPLC (Agilent Technologies, Inc., Santa Clara, CA) with an anion exchange column (PRP-X100, 10 µm, 250 × 4.1 mm, Hamilton, Reno, NV) and guard cartridge. The mobile phase was 50 mM ammonium carbonate (pH 9) with 4% (*v/v*) methanol at a flow rate of 1.0 mL/min. Column temperature was maintained at 30 °C and samples were kept at 4 °C in a thermally controlled auto sampler. An Agilent 7500ce ICP-MS with a Conikal nebulizer (Glass Expansion) was used as the detector. The operating and acquisition parameters were the same as for total arsenic analysis.

NIST SRM2670a Toxic Elements in Urine (National Institute of Standards and Technology, Gaithersburg, MD, USA) was used for QA/QC purposes. The standard reference material was run in duplicate with every set of 20 samples. For analysis of total arsenic, the percent recovery was between 96.7% and 107.5% with a coefficient of variance (COV) of 2.7. For arsenic speciation, the percent recovery was between 93.5% and 97.4% with a COV of 1.7.

Results included concentrations of total arsenic, the inorganic species: As^3^ and As^5^, and the organic species: MMA^5^, and DMA^5^. Analysis of the arsenic species MMA^3^ and DMA^3^ was not possible due to their unstable nature. The detection limits for the arsenic species are as follows: total arsenic 0.1 µg/L, As^3^ 0.12 µg/L, As^5^ 0.21 µg/L, MMA^5^ 0.12 µg/L, and DMA^5^ 0.12 µg/L.

### 2.8. Estimation of Arsenic Exposure in Water and Beverage

Consumption of water and other beverages was reported in liters per day (L/day) from the 24 h dietary recall. The interviewer asked participants to report all water consumed, regardless of location or source, on the dietary recall. To determine exposure from drinking water, we assumed water consumed outside of the home would have similar arsenic water concentrations as those in the home. This is a reasonable assumption since homes are generally on the same water system as other local venues (*i.e.*, work, school, restaurants, *etc.*) or water is drawn from the same aquifer in most cases. Of the 465 people in the analysis, there were 362 people with water intake volumes recorded on the 24 h dietary recall and 103 people for whom total water volume consumed for the day was not recorded. A single imputation approach was used to estimate the total water volume consumed in a 24 h period for those without a total water volume. For participants lacking a consumption volume, the average total water consumption was substituted based on age, gender, and recruitment site categories. The age categories included: 18–29, 30–39, 40–49, 50–59, 60–69, 70+. The recruitment site categories included Arizona, Hermosillo, and the Yaqui Valley. The imputed water consumption volumes among the categories are not shown but as an example, a female participant from Arizona in her 30 s who had a missing total water consumption volume would be estimated to have consumed 1.02 L of water for the day reflecting the mean consumption of others in this category.

Every water source identified as consumed in the home, including bottled water, was tested for total arsenic. Participants were asked to report usual consumption for each water source. Consumption categories included frequently, moderately, rarely, and never. A weighted average based on these frequencies was used to determine total volume of water consumed from the individual sources. A frequency value was assigned for consumption (e.g., 3 for frequent use, 2 for moderate use, 1 for rare use, and 0 for no use). For example, if a participant drank from three water sources, the first of which was consumed “frequently” (3), the second “moderately” (2), and the third “frequently” (3), the water sources could be assigned proportions of 3/8, 2/8, and 3/8 respectively with the denominator being the sum of the values. The fractions were multiplied by the total daily water volume resulting in a volume for each water source. The arsenic concentration per source was then multiplied by the amount of water consumed yielding the amount of arsenic consumed in a 24 h period (µg/day) per source. The arsenic intake from each water source was then summed to estimate the total arsenic intake from all water sources consumed in the home for the day.

Total arsenic intake from other beverages was calculated by multiplying arsenic concentration of specific beverages as reported in the literature by the reported volume from the 24 h recall. The arsenic intake from each beverage was then summed to estimate the total arsenic intake from all beverages consumed for the day.

### 2.9. Analysis Strategy

Of the 487 participants, 19 people were excluded in this analysis due to inadequate information regarding water consumption and three people were excluded for consumption reports exceeding 10 L of water a day.

Urinary total arsenic, inorganic arsenic (expressed as the sum of As^3^ and As^5^), and sum of species (expressed as the sum of As^3^, As^5^, MMA, and DMA) were reported as continuous variables in micrograms per liter. The relative distributions of arsenic species were reported as the percent MMA (MMA over sum of species) and the DMA/MMA ratio. Half of the detection limit was substituted in instances where arsenic concentration was below the detection level. Urinary inorganic arsenic, sum of species, and total arsenic concentrations were adjusted for specific gravity using the formula reported by Nermell *et al.* using the specific gravity population mean [16]. The distributions of urinary total arsenic, sum of species, inorganic As, DMA/MMA ratio, arsenic concentration consumed from water, and arsenic concentration consumed from all beverages were right skewed and were natural log transformed.

Summary statistics were generated for urinary total arsenic, inorganic arsenic, DMA/ MMA ratio, and percent MMA. A Kruskal-Wallis test was used to detect any differences in the various urinary arsenic measurements between recruitment sites. Random effect models were used to evaluate the association between the concentration of arsenic in urine and the estimated amount of arsenic consumed from water and other beverages. The random effect in all models was defined as household nested within study site. A random effect model was selected to account for the variance within households and across study sites. Other variables were considered in the statistical analyses with their potential to be associated with both the concentration of total arsenic in urine and the estimated amount of arsenic consumed from drinking water. These variables included: age in years (continuous), gender (male, female), ethnicity (Arizona Hispanic, Arizona non-Hispanic, Mexicano), and current smoking status (current smoker, not current smoker). Ten models were generated. The predictor in models 1–5 was arsenic concentration consumed from water (log arsenic values). The predictor in models 6–10 was log arsenic concentration consumed from all beverages. Each set of five models, adjusted for confounders, contained one of the following as the dependent variable for urinary arsenic concentration: log total arsenic, log inorganic arsenic, log sum of species, log DMA/MMA ratio, and percent MMA. All statistical analyses were completed using SAS (version 9.2; SAS institute, Cary, NC).

## 3. Results

### 3.1. Household Recruitment

The goal was to recruit at least 50 households from each neighborhood/community in Mexico and 100 households in Arizona (25 per community). Household participation by community ranged from 18% to 30% in Arizona, as shown in Table 1. Household participation by mail in New River was 20%. In contrast, household participation rates were greater in all Mexican sites ranging from 43% to 93% (Table 2) with participation lower in the more urban neighborhoods of Hermosillo. At least one person per household was asked to participate at all study sites. The mean number of interviewed people per household was 1.7 in Arizona, 1.1 in Hermosillo and 1.5 in Yaqui Valley.

**Table 1:** Household Participation Rates among Arizona Residents by Location.

	Arizona			
Characteristic	Ajo	New River	San Manuel	Tucson
List-assisted dialing				
Phone Numbers Dialed	692	699	974	413
Households Recruited	25	29	31	47
Participants Recruited	31	77	49	68
Households that Refused to Participate	57	75	142	143
Household Unavailable on Day of Sampling	7	4	6	0
Only an Answering Machine	60	215	136	0
Household Never Answered the Phone	41	89	118	116
Business/Fax Machine	64	104	69	19
Disconnected Phone Numbers	438	183	472	88
Household Participation Rate *	30%	28%	18%	25%
Mailing				
Households Mailed a Brochure	-	101	-	-
Households Recruited from Mailing	-	20	-	-
Household Mailing Participation Rate ^†^	-	20%	-	-

* Household Participation Rate = Households recruited/(Households recruited + Households that refused to participate); ^†^ Household mailing participation rate = Households recruited from mailing/Households mailed a brochure

**Table 2:** Household Participation Rates from Door-to-Door Recruitment in Sonora, Mexico by Location.

	Hermosillo		Yaqui Valley	
Characteristic	Community 1	Community 2	Guadalupe Victoria	Tobarito
Households Selected for Recruitment	107	122	62	68
Households Recruited	50	50	52	50
Participants Recruited	55	56	61	90
Households that Refused to Participate	41	59	4	8
No Eligible Participant in the Household	3	1	2	2
No Response from Household	13	12	3	8
Household Participation Rate *	55%	46%	93%	86%

* Household Participation Rate = Households recruited/(Households recruited + Households that refused to participate)

### 3.2. Participant Characteristics and Water Consumption

Summary statistics for participant characteristics are presented in Table 3. More females than males participated across the three recruitment sites: Arizona 56.9%, Hermosillo 73.2%, Yaqui Valley 69.8%. The median age of the participants from Arizona was 56.5 years compared to 39.5 years from Hermosillo and 44.0 years from Yaqui Valley. Hispanics accounted for 24% of the participants from Arizona. There were significant differences (*p* < 0.001) in the current smoking rates across the populations (16.7% from Arizona, 30.3% from Hermosillo, and 11.1% from Yaqui Valley).

**Table 3:** Demographic and Lifestyle Characteristics of BAsES Participants by Location.

Characteristic	Arizona	Hermosillo	Yaqui Valley	*p*-value
Participants ( *n*)	218	108	139	
Age in years				< 0.001 *
Mean (s.d.)	55.3 (15.3)	41.5 (13.9)	47.0 (16.4)	
Median	56.5	39.5	44.0	
Gender, *n* (%)				< 0.001^†^
Male	94 (43.1)	29 (26.7)	42 (30.3)	
Female	124 (56.9)	79 (73.2)	97 (69.8)	
Ethnicity, *n* (%)				-
Arizona Hispanic	52 (23.9)	0	0	
Arizona Non-Hispanic	166 (76.1)	0	0	
Mexicano	0	108	139	
Current smoker, *n* (%)				< 0.001^†^
Yes	33 (16.7)	30 (30.3)	15 (11.1)	
No	165 (83.3)	69 (69.7)	120 (88.9)	
Unknown	20	9	4	
Self-reported estimated fluid intake from the 24-hour dietary recall (L/day)				
Drinking water				< 0.001*
Mean (s.d.)	1.67 (1.20)	0.44 (0.36)	0.24 (0.17)	
Median	1.43	0.38	0.24	
*n*	218	108	139	
Non-water beverages				< 0.001*
Mean (s.d.)	1.77 (1.25)	0.92 (0.85)	0.58 (0.32)	
Median	1.39	0.79	0.52	
*n*	214	102	130	
Total beverage consumption				< 0.001*
Mean (s.d.)	3.51 (1.89)	1.31 (0.93)	0.79 (0.36)	
Median	2.88	1.18	0.75	
*n*	218	108	139	

L = Liters; s.d. = standard deviation; * Kruskall-Wallis test of association across location; ^†^ Chi-square test of significance across location.

Median water consumption reported in the 24 h dietary recall was 1.43 L/day among Arizona participants, 0.38 L/day among Hermosillo participants, and 0.24 L/day among Yaqui Valley participants (*p* < 0.001). The median non-water beverage consumption was greatest among Arizona residents at 1.39 L/day and was significantly different among the three sites (*p* < 0.001). Median total fluid intake was significantly different (*p* < 0.001) between participants from Arizona (2.88 L/day), from Hermosillo (1.18 L/day), and from the Yaqui Valley (0.75 L/day).

### 3.3. Distribution of Arsenic in Water and Beverages

Table 4 contains the concentration of arsenic in unfiltered tap water coming into the home by community. The highest average arsenic concentration was found in the community of New River (98.9 µg/L). The lowest median arsenic concentration was found in Tucson (3.9 µg/L). Also shown in the table are the estimated total arsenic concentrations consumed from water and other beverages by community. Median total arsenic consumed from water by participants in Arizona ranged from 1.7 to 14.1 µg/day, compared to 0.6 to 3.4 µg/day for participants from Mexico. Estimated total arsenic intake via water was greatest among participants from New River and least among participants from Tobarito, Mexico. When examining estimated arsenic intake from all beverages, participants in Mexico had lower daily consumption rates than most participants from Arizona. This corresponds to participants in Mexico reporting less volume per day from beverages leading to a lower estimated intake of As through beverages.

**Table 4:** Arsenic Levels in Water and Estimated Total Arsenic Intake from Drinking Water and Other Beverages during 24 h by Recruitment Site.

	Arizona				Hermosillo		Yaqui Valley	
	Ajo	New River	San Manuel	Tucson	Community 1	Community 2	Guadalupe Victoria	Tobarito
**As levels from an unfiltered water source by household (µg/L)**								
Households (*n*)	25	48	31	47	48	49	48	37
As concentration								
Mean (s.d.)	9.7 (26.2)	98.9 (198.5)	6.9 (2.2)	4.3 (6.6)	24.5 (7.6)	8.6 (2.3)	4.8 (2.5)	7.6 (7.7)
Median	4.2	22.1	6.9	3.9	26.3	9.0	4.1	4.0
**Estimated total arsenic intake (µg/day)**								
Participants (*n*)	31	71	48	68	54	54	52	87
From drinking water								
Mean (s.d.)	5.2 (4.3)	166.0 (468.4)	9.0 (2.5)	4.1 (5.8)	5.5 (6.8)	2.4 (2.5)	1.9 (1.5)	1.1 (1.5)
Median	4.2	14.1	1.7	2.8	3.4	1.7	1.6	0.6
From total beverage consumption								
						
Mean (s.d.)	9.3 (7.3)	176.3 (467.1)	13.0 (9.9)	8.9 (9.3)	9.4 (16.2)	5.4 (4.6)	3.9 (2.7)	3.9 (6.6)
Median	8.3	27.9	11.0	6.1	5.4	4.7	3.0	2.1

s.d. = standard deviation.

### 3.4. Distribution of Arsenic in Urine

Table 5 presents the distributions of urinary arsenic concentrations adjusted for specific gravity by community. The greatest concentrations of total and inorganic urinary arsenic were among participants in New River. However, except for this one Arizona community, median urinary total As concentration among Arizona participants (ranged from 1.2 to 2.0 µg/L) was less than the median urinary inorganic As concentration for Mexican participants (range 2.5 to 6.2 µg/L). Among Arizonans, the average sum of species was highest among those from New River (39.9 µg/L). The participants from Arizona communities, except those in New River, had a lower median sum of species level than participants from Mexico communities. The sum of species was not significantly different between communities in the Yaqui Valley, but it was significantly different between the communities in Hermosillo (*p* < 0.001). There were no differences in levels of DMA/MMA or % MMA by community. 

**Table 5:** Urinary Arsenic Concentration Adjusted for Specific Gravity among BAsES Participants by Recruitment Site.

	Arizona				Hermosillo		Yaqui Valley	
	Ajo	New River	San Manuel	Tucson	Community 1	Community 2	Guadalupe Victoria	Tobarito
Participants ( *n*)	31	71	48	68	54	54	52	87
Total As (µg/L)								
Mean (s.d.)	35.3 (27.2)	121.7 (221.4)	42.2 (19.6)	47.7 (94.0)	61.6 (43.3)	110.0 (89.9)	119.3 (131.8)	93.0 (108.7)
Median	25.9	33.0	36.3	25.4	50.7	79.9	94.3	64.6
Inorganic As (µg/L)								
Mean (s.d.)	2.9 (3.6)	10.2 (28.5)	2.3 (2.9)	1.9 (3.0)	3.5 (3.1)	7.4 (5.6)	6.7 (4.4)	5.7 (5.8)
Median	2.0	1.7	1.4	1.2	2.5	6.2	6.0	3.7
Sum of Species (µg/L)								
Mean (s.d.)	8.6 (4.2)	39.9 (104.2)	8.1 (5.4)	7.7 (7.0)	13.5 (6.9)	26.1 (25.0)	21.9 (11.4)	21.3 (15.8)
Median	8.5	7.4	6.0	6.3	12.2	20.5	18.8	17.2
DMA/MMA ratio								
Mean (s.d.)	7.1 (4.3)	7.2 (4.2)	7.8 (4.2)	7.6 (3.8)	7.6 (4.5)	8.2 (5.3)	7.8 (4.1)	7.8 (4.5)
Median	5.9	6.2	7.1	6.8	6.3	6.8	6.7	6.7
Percent MMA								
Mean (s.d.)	12.1 (4.8)	12.8 (4.8)	11.6 (4.6)	11.8 (3.9)	11.9 (3.9)	11.1 (4.1)	11.2 (3.8)	11.4 (3.5)
Median	12.3	12.5	10.7	11.7	12.1	11.1	10.5	11.0

s.d. = standard deviation; inorganic As = As^3^ + As^5^; Sum of Species = As^3^ + As^5^ + MMA + DMA; Percent MMA = (MMA/Sum of Species)*100.

### 3.5. Associations Between Arsenic Exposure and Urinary Arsenic Concentrations

Table 6 presents results of ten random effect models evaluating the association between concentrations of urinary as species and estimated As exposure from water and other beverages. The estimated arsenic intake from water consumption had a statistically significant association with each of the five urinary As variables. Due to the independent and dependent variables being log transformed, inferences of the model estimates come from the interpretations found in *Regression Methods in Biostatistics* [17]. We can infer from model 1 that there is a 7% increase in total urinary arsenic for a 1% increase in arsenic consumption from water (*p* < 0.001), from model 2 that there is a 12% increase in urinary inorganic arsenic for a 1% increase in arsenic consumption from water (*p* < 0.001), and from model 3 that there is a 9.8% increase in urinary sum of species for a 1% increase in arsenic consumption from water. In contrast, no statistically significant association was found between estimated arsenic intake from all beverages and either urinary DMA/MMA ratio or urinary percent MMA. A significant association was found between estimated arsenic intake from all beverages with urinary total As, inorganic As, and sum of species.

**Table 6:** Random Effect Models Depicting the Association between Urinary Metabolite Concentrations and Arsenic Consumed from Drinking Water and Beverages *.

Dependent variable	Estimate of Log As consumed from water ± s.e.	*p*-value
Model 1: Urinary Log (Total As)	0.074 ± 0.020	<0.001
Model 2: Urinary Log (Inorganic As)	0.123 ± 0.022	<0.001
Model 3: Urinary Log (Sum of Species)	0.098 ± 0.021	<0.001
Model 4: Urinary Log (DMA/MMA ratio)	−0.032 ± 0.012	0.007
Model 5: Urinary (Percent MMA)	0.252 ± 0.103	0.016
	**Estimate of Log As consumed from all beverages ± s.e.**	***p*-value**
Model 6: Urinary Log (Total As)	0.143 ± 0.032	<0.001
Model 7: Urinary Log (Inorganic As)	0.234 ± 0.036	<0.001
Model 8: Urinary Log (Sum of Species)	0.203 ± 0.034	<0.001
Model 9: Urinary Log (DMA/MMA ratio)	−0.029 ± 0.019	0.130
Model 10: Urinary (Percent MMA)	0.214 ± 0.170	0.213

* The random effect was the variable household nested within study site. Adjusted for age, gender, current smoking status, and ethnicity; s.e. = standard error; inorganic As = As^3^ + As^5^; Sum of Species = As^3^ + As^5^ + MMA + DMA; Percent MMA = (MMA/Sum of Species)*100.

## 4. Discussion

This paper describes the relationship between urinary arsenic concentrations and estimated arsenic intake from beverages from a cross-sectional survey of community-based residents living in southern Arizona and northern Mexico. Residents from the rural communities in Yaqui Valley and rural areas in Arizona were more likely to participate than residents living in urban areas, and household response rates were greater in Mexico than in Arizona. Anecdotally, participants from Mexico liked the attention received from a study conducted by the local university. 

Strikingly, the estimated volume of all beverages consumed among participants from Arizona was nearly four times greater than the amount consumed by participants in Mexico. This difference reflected either reduced consumption of beverages by Mexican participants compared to Arizona participants (0.75 L *vs.* 2.9 L), a misrepresentation of the amount of beverages consumed by Mexicans, or over reporting of beverages consumed by Arizona participants. A reported water volume that was incorrect would alter the estimated arsenic level consumed from water for the day.

The median volume of water consumed by participants from Arizona (1.4 L/day) is similar to the reported consumption noted in the literature [18]. The largest database containing water consumption comes from the National Health and Nutrition Examination Survey (NHANES) from 1999 to 2001 [18]. Nearly 88% of participants reported drinking an average amount of 1.53 L/day, while 12% drank no water [19]. In a pilot study in Canada, researchers found that 56% of the population drank bottled or filtered water the previous day with an overall water consumption rate of 1.6 L/day [20]. 

Median estimated arsenic intake from water in Arizona communities ranged from 1.7 to 14.1 µg/day compared to 0.6 to 3.4 µg/day in Mexico communities. In contrast to the estimated exposures, the median urinary inorganic arsenic concentration was lowest among participants from Tucson 1.2 (µg/L) and highest among participants from Hermosillo (6.2 µg/L). Median urinary total arsenic concentration was lowest in Tucson participants (25.4 µg/L) and highest among Yaqui Valley participants (94.3 µg/L). The discrepancy between urinary inorganic arsenic concentration and total arsenic concentration among participants could potentially be due to the ingestion of organic forms of arsenic.

Table 4 shows the median arsenic concentration of the drinking water coming into the home and is similar among several of the study sites. If the median volume of water consumed for the day was similar among participants from each study site, then we would expect the intake of arsenic from water between the study sites to be similar. However, we find that the estimated arsenic intake from water is lower among several of the communities in Mexico. It is possible that the total water intake from Mexico participants is underestimated thus resulting in an underestimate of total arsenic intake. Further analysis into reporting of intake volumes of water and beverages is under review.

Strengths of this study include the use of standardized methodologies for sample collection, administration of questionnaires, and data entry. A centralized laboratory was used to analyze urine and water samples from participants as well as centralized analysis of the 24 h dietary recalls.

Limitations in this study include potential selection bias. Participants were mostly female. This could be addressed in future studies conducted in Mexico by recruiting during the evening and on weekends. Other demographic characteristics in the U.S. such as age, ethnicity and race were comparable to percentages found in the Arizona census.

An additional limitation is that studies reporting arsenic concentrations in foods and beverages have primarily been conducted among products sold in the United States. In this analysis, the arsenic concentration in beverages are coming from the paper by Schoof *et al.* [15] These data sources are limited by the types of foods and beverages being analyzed as well as the quantity being analyzed. A single arsenic concentration for one product is being applied to a wide array of beverages. Furthermore, these databases may not represent the food products in subregions of the U.S. or outside the U.S. Few data are available on the concentration of arsenic in beverages sold in Mexico which may resulted in an over or under estimation of the arsenic concentration among beverages consumed by Mexican participants.

Furthermore, in this analysis it was assumed that the arsenic concentrations in urine were attributed to the water and beverages consumed the previous day. Urine was collected on the day of the interview and given the study resources and known recall bias we could only collect a dietary recall for the day prior the interview. It has been reported that people may be able to recall the preceding 24 h of food and beverage consumption with interviewer prompts [21]. It is difficult for people to recall amounts ingested for 2–3 days. Using a prospective design, investigators could monitor food and beverage consumption for 2–3 days prior to collection of the urine sample.

Reported water consumption for the day was missing from 28% of the 24 h dietary recalls. This was due to the interviewers not prompting for water consumption during the interview. We also used qualitative terms in this study to identify amount of water consumed per source based on the total intake of water from the 24 h dietary recall. The Arizona Diet, Behavior and Quality of Life Assessment Lab suggested a conservative scoring system to assign volumes to each water source using a 1 point difference to separate frequent, moderate, and rare consumption. By using a conservative scoring system we avoided over estimating arsenic consumption from water intake. Future studies exploring contaminants in drinking and cooking water via a 24 h dietary recall should specifically ask participants about the volume of water consumed per source avoiding the need to estimate the volume. 

Communities in BAsES were in part selected for their groundwater arsenic concentration so that there would be communities with a higher or lower arsenic concentration in the household tap water. The two communities selected in Hermosillo display this relationship. One community had a median tap water arsenic concentration of 26.3 µg/L while the other was at 9.0 µg/L. This contrast was not seen in the two communities of the Yaqui Valley. In Tobarito, the community water source was unavailable and residents retrieved their water in bottles from a well in a nearby community. This change in water source was unknown until after the study had been completed. As a result, fewer people were exposed to elevated concentrations of arsenic via drinking water in the Yaqui Valley. 

The statistical model used in this analysis was based on a strategy to describe the association between estimated arsenic intake from water and arsenic output in urine. Among participants in the U.S., the greatest urinary arsenic concentrations were seen in geographic locations with the greatest concentration of arsenic in the drinking water. However; among the participants from Mexico, elevated urinary arsenic levels were present among individuals consuming lower concentrations of arsenic in the drinking water at home. It may be that the arsenic presenting in the urine was consumed from water sources with arsenic concentrations different from arsenic concentrations in water sources from the home or from food. Analysis are ongoing to develop predictive models that include a detailed examination of the potential impact of beverages and other food items reported in the 24 h dietary recall in the prediction of urinary arsenic concentration. Furthermore, studies should be undertaken to examine beverage consumption patterns among the various ethnicities and the role other metals (e.g., selenium or cadmium) may have on the impact of As metabolism.

## 5. Conclusion

BAsES enhanced a binational collaboration between universities in Mexico and the U.S., built research capacity, and enabled collaborations in a region experiencing rapid changes, within the same desert environment. Standardized questionnaires and recruitment methods were utilized among all recruitment sites. A standardized and common laboratory for testing total, organic, and inorganic As was utilized. Estimated arsenic concentrations consumed from water and other beverages were found to be greater among Arizonans, yet participants in Mexico had greater urinary inorganic arsenic concentrations.
